# Associations of Environmental Modifications and Collaborative Care Environments with Positive Health in Families of Children with Medical Complexity: A Secondary Analysis

**DOI:** 10.3390/nursrep16060192

**Published:** 2026-06-05

**Authors:** Yumi Mizuochi, Yukako Shigematsu, Yoshitomo Fukuura, Kyoko Miwa

**Affiliations:** 1 Department of Nursing, School of Medicine, Kurume University, 777-1 Higashikushiharamachi, Kurume 830-0003, Japan; shigematsu_yukako@kurume-u.ac.jp; 2 Faculty of Nursing, Department of Nursing, Yasuda Women’s University, 6-13-1 Yasuhigashi, Asaminami-ku, Hiroshima 731-0153, Japan; fukura-y@yasuda-u.ac.jp; 3 Graduate School of Nursing, Osaka Metropolitan University, 1-5-17 Asahimachi, Abeno-ku, Osaka 545-0051, Japan; kmiwa@omu.ac.jp

**Keywords:** children with medical complexity, environmental modifications, family health, home-based care

## Abstract

**Background/Objectives**: Improved survival rates have led to an increase in the number of children with medical complexity (CMC) receiving home-based care. However, there is a lack of clarity regarding the relationships among collaborative environments, environmental modifications, and positive family health among families of CMC in daily living settings. This study aimed to examine these relationships and identify their associated factors. **Methods**: This study was a secondary analysis of data derived from a self-administered questionnaire that was distributed to the families of CMC with experience in organizing the care environment. Ninety responses were included in the study, and regression analyses were performed using complete cases (*n* = 41–63). **Results**: Family-led environmental modifications (β = 0.670, *p* < 0.001) and physical environmental modifications (β = 0.679, *p* = 0.015) were positively associated with the collaborative environment, whereas professional-facilitated family-led environmental modifications were negatively associated with the collaborative environment (β = −0.775, *p* = 0.009). Regarding positive health in families, family-led environmental modifications (β = 0.487, *p* = 0.018), environmental modifications for care improvement (β = 0.597, *p* = 0.031), pre-modification family well-being (β = 0.464, *p* < 0.001), and the presence of someone to consult (β = 0.330, *p* = 0.011) were significantly associated with positive health in families. Because this study employed a cross-sectional design, causal relationships cannot be inferred. **Conclusions**: Collaborative environments in daily living settings may be associated with family involvement, physical environmental conditions, and professional engagement. Healthcare professionals may support family autonomy and participation in environmental modification processes.

## 1. Introduction

Advances in perinatal medicine have enabled the survival of extremely preterm infants who were previously unlikely to survive [1,2,3]. Consequently, more children with severe chronic conditions and those requiring ongoing medical care, referred to as children with medical complexity (CMC), currently receive care in home and community settings [4]. CMC generally refers to children with chronic and complex health conditions who often require substantial healthcare support, technology dependence, and long-term home and community-based care.

In these settings, care for CMC is provided mainly by families [5,6]. Families experience substantial physical, psychological, and social burdens due to continuous caregiving [7,8]. Physically, these caregivers often experience fatigue and musculoskeletal pain related to frequent medical procedures and care tasks and report a higher caregiving burden than those caring for children without complex needs [9]. Psychologically, anxiety, depression, and sleep disturbances are common, and their mental health tends to be poorer than that of the general population [10]. Socially, caregiving demands limit employment and social participation and may lead to isolation that affects the entire family, including siblings and partners [11]. Additionally, when support systems are insufficient, families must compensate for gaps in healthcare and welfare services, resulting in increased time and financial burdens.

To support stable living for CMC, it is important to optimize the family’s living environment. The care environment in daily settings includes physical arrangements, such as medical equipment and housing modifications, as well as access to healthcare and welfare services and a stable foundation that enables safe caregiving [12,13]. These elements form the basis for maintaining and improving the quality of life of children and their families. In this study, the “care environment in daily living settings” refers to a comprehensive environment that includes a safe and comfortable physical environment; a collaborative environment between families and professionals; a service environment that ensures access to necessary services; and a supportive community environment [13].

Collaborative relationships between families and professionals are essential for the effective functioning of this environment. While care for CMC requires specialized knowledge, most daily care is provided by families [5]. Sharing information and involving families in decision-making have been associated with a reduced caregiver burden and better quality of life [14,15]. Professionals provide medical expertise, while families contribute knowledge of the child’s daily life and context. Integrating these perspectives is important for improving care.

To reduce the caregiving burden, it is also important for families to take an active role in organizing their living environments. Family-led environmental adjustments may support the care environment and are associated with better family well-being [13,16]. In particular, collaboration with professionals may contribute to reducing family burden [16].

In the context of chronic conditions, health is increasingly understood as the ability to adapt to and manage daily life, rather than a fixed state [17]. In this study, “positive health in families” refers to families’ perceived ability to adapt, self-manage, and maintain well-being while responding to the physical, psychological, and social challenges of caring for a child with medical complexity [18,19].

Recent studies have highlighted the role of psychosocial resources, such as resilience and social support, in reducing caregiver stress and improving quality of life [20,21]. These resources are considered key factors that support families’ adaptive capacity and sustain positive health in challenging caregiving contexts.

However, most previous studies on families of CMC have focused on outcomes such as caregiver burden and health-related quality of life [14,22]. Limited evidence exists regarding how environmental modifications and collaborative care environments are associated with positive health among families of children with medical complexity in daily living settings. This lack of evidence limits understanding of how positive health in families can be supported through environmental and collaborative care factors in home-based care settings.

To address this gap, we conducted a secondary analysis of data partially reported in our previous study [16]. The previous study examined collaborative care environments among multiple stakeholder groups and did not include positive health as an outcome. In contrast, the present study is a family-only secondary analysis focusing specifically on families of children with medical complexity. This study newly examines the associations among environmental modifications, collaborative care environments, and positive health in families, which was not analyzed in the previous publication. In addition, the present study applies regression models specifically targeting positive health as the dependent variable, which represents a different analytical focus from the prior study.

Therefore, building on this secondary dataset, this study aimed to examine the relationships among collaboration with professionals, environmental adjustments, and positive health in families among families of CMC and identify factors associated with these relationships.

## 2. Materials and Methods

### 2.1. Study Design

This study employed a cross-sectional observational design, using a self-administered questionnaire. This study is a secondary analysis of data derived from a previously conducted nationwide survey [16]. Whereas the previous study examined collaborative care environments among multiple stakeholder groups, the present study focused specifically on family respondents and examined the relationships between environmental modifications, collaborative care environments, and positive family health from the family perspective.

This study was conducted and reported in accordance with the STROBE statement.

### 2.2. Ethical Considerations

This study was conducted in accordance with the Declaration of Helsinki. This study was approved by the Ethics Committee of Kurume University (approval number: 24109). Facility representatives were provided with written information outlining the study’s purpose, methods, ethical considerations, and plans for dissemination. Questionnaires and request forms were distributed to the participants through these representatives.

To ensure anonymity, the questionnaires were unsigned, and no identifying information was included. Participants were informed that participation was voluntary and that refusal would not result in any disadvantages. Consent was obtained by checking a consent box at the beginning of the questionnaire. Participants were also informed that the results would be published and that, owing to pseudonymization, the submitted questionnaires could not be withdrawn or deleted.

### 2.3. Definition of Terms

Daily living settings: Places where children requiring medical care live with their families.

Care environment: Environment surrounding daily life and care, consisting of the following:

1. Physical environment: Arrangement of medical equipment, beds, and supplies in the child’s living space.
2. Collaborative environment: Collaboration between families and professionals to support CMC and their families.
3. Community environment: Community in which the children live with their families.
4. Service environment: Services that support the daily lives of the children and their families.

Environmental modifications: Activities to structure or adjust the environment of children and their families in daily living settings. These included activities undertaken by families alone, those conducted jointly by families and professionals, and those led by professionals.

1. Physical environmental modifications: Environmental modifications related to arranging physical spaces, supplies, and preparedness to support the child’s daily care, safety, and development in living settings.
2. Family-led environmental modifications: Environmental modifications primarily initiated and directed by families based on their own values, preferences, daily routines, and childrearing priorities.
3. Family-led environmental modifications facilitated by the professionals: Environmental modifications in which families remained the primary decision-makers while receiving informational, consultative, coordinative, or facilitative support from professionals.
4. Service environmental modifications: Environmental modifications related to accessing, coordinating, and optimizing health, welfare, and community services necessary for family life and childrearing.
5. Community environmental modifications: Environmental modifications aimed at promoting the child’s and family’s connection, participation, and inclusion within the local community.
6. Care improvement modifications: Environmental modifications focused on improving the quality, responsiveness, and family-centeredness of care and support provided to children and families.

Family: Parents, grandparents, and siblings of the child (including co-resident and non-co-resident family members). Professionals: Medical and welfare professionals involved in environmental modifications in daily living settings.

### 2.4. Sample Size Considerations

This study was a secondary analysis of an existing nationwide survey [16], and the sample size was determined by the number of eligible family respondents available in the original dataset. Therefore, no a priori sample size calculation was conducted. The present analyses were considered exploratory.

### 2.5. Participants and Data Collection

Participants were families of children aged 0–18 years with CMC who required ongoing medical care, healthcare support, and environmental modifications in daily living settings. CMC generally includes children with chronic and complex health conditions associated with technology dependence, functional limitations, and/or the need for home nursing or welfare services. The participating children had various chronic health conditions, including neurological, gastrointestinal, respiratory, and excretory disorders. Examples of ongoing medical care included ventilator management, tracheostomy care, tube feeding management, stoma care, clean intermittent catheterization, inhalation therapy, and suctioning.

Eligible respondents were family members involved in the child’s daily care and environmental organization. Responses from families who had not implemented environmental modifications or who provided incomplete responses or missing data on variables included in the analyses were excluded using complete-case analysis.

From September 2024 to March 2025, prefectures across Japan were selected based on geographical region and population size. A total of 500 home-visit nursing stations, 150 consultation support offices, and 150 child development support and after-school day service facilities for CMC were then selected from these prefectures. One family questionnaire was distributed to each home-visit nursing station and consultation support office, and two family questionnaires were distributed to each child development support and after-school day service facility for CMC. In addition, 26 family associations were invited to distribute questionnaires to eligible families.

Seventeen family associations agreed to participate and requested a web-based survey option. Therefore, family responses were collected using both postal questionnaires and an online survey via Google Forms.

Because recruitment was conducted indirectly through facilities and family associations, the exact number of eligible families who received the questionnaires or survey links could not be determined.

Among the returned questionnaires/responses, 93.8% met the eligibility criteria and were included in the analysis. The participant recruitment and analysis processes are shown in Supplementary Figure S1.

### 2.6. Instruments

#### 2.6.1. Questionnaire Items

##### Characteristics of Families

For families, the basic attributes included the primary caregiver, presence and number of siblings, employment status of the primary caregiver, presence of interactions with other family members, presence of a person to consult, and family relationships.

Environmental modification items included the context of environmental modification, child’s health status, child’s signs and responses, child’s expression of intent, and background of environmental modifications (availability of desired services, ease of service use, information about needed services, people with whom the family communicates, people the family can ask for help, and people with whom the family can share private matters).

##### Items Related to the Care Environment

Items related to the care environment were extracted from the conceptual framework of a previous review [13]. The questionnaire items were newly developed for this study based on a concept analysis of care environments in daily living settings. Thirty-five items inquired about the pre- and post-environmental modifications. Responses were rated on a 7-point Likert scale: very well organized (6), well organized (5), somewhat organized (4), neither well nor poorly organized (3), not very well organized (2), hardly organized (1), and not organized at all (0). Additionally, for aspects unrelated to environmental modifications, the option ‘not applicable’ was provided and responses were treated as missing values when selected.

The complete list of items is provided in Table S1 (Supplementary Materials).

##### Items Related to Environmental Modifications

Items related to environmental modifications were extracted from interviews with 20 professionals involved in environmental modifications in the daily living settings of CMC, including hospital nurses, home-visit nurses, consultation support specialists, nurses working in child development support and after-school day services, medical social workers, and public health nurses. These questionnaire items were newly developed based on qualitative interview research involving professionals who support CMC and their families. Overall, 48 items assessed the extent to which environmental modifications were implemented. Responses were rated on a 5-point Likert scale: implemented extensively (5), implemented (4), somewhat implemented (3), rarely implemented (2), and not implemented (1). Additional options of ‘not necessary’ and ‘don’t know’ were provided, and the responses were treated as missing values when selected. Of the 48 items, 19 were related to modifications performed by families, nine were related to modifications performed jointly by families and professionals, and 20 were related to modifications performed by professionals.

The complete list of items is provided in Table S2 (Supplementary Materials).

##### Items Related to Family Well-Being

Items related to family well-being were developed based on a literature review of previous studies [10,23,24]. The questionnaire items were newly developed for this study based on the concept analysis of care environments in daily living settings, in which patient and family well-being were identified as key outcomes, as well as a literature review related to family well-being among families of children with medical complexity. Thirteen items assessed family well-being before and after environmental modifications. Responses were rated on a 7-point Likert scale: strongly agree (6), agree (5), somewhat agree (4), neither agree nor disagree (3), somewhat disagree (2), disagree (1), and strongly disagree (0). Total scores ranged from 0 to 78, with higher scores indicating greater family well-being. Cases with missing values were excluded from the analysis.

##### Items Related to Positive Health in Families

Items related to positive health in families were developed based on a literature review of previous studies [17,25,26]. The questionnaire items were newly developed for this study based on Huber’s Positive Health framework and a literature review related to positive health in families. Ten items assessed positive health in families and were answered only by family members. Responses were rated on a 5-point Likert scale: strongly able (5), able (4), somewhat able (3), rarely able (2), and not able at all (1). Total scores ranged from 10 to 50, with higher scores indicating greater positive health in families. Responses with missing values were excluded from the analysis.

The psychometric properties of all study instruments are presented in Table S3 (Supplementary Materials). For scales with high Cronbach’s alpha coefficients, item content was additionally reviewed to assess potential redundancy; however, no substantial overlap in item content was identified.

### 2.7. Data Analysis

Statistical analyses were conducted using IBM SPSS Statistics, version 30 (IBM Corp., Armonk, NY, USA). For each analysis, participants with missing data on any of the variables included were excluded from that analysis, and only complete cases were used. A *p*-value of less than 0.05 was considered statistically significant for all analyses, and all tests were two-sided.

Multiple regression analyses were conducted to examine environmental modification factors associated with collaborative care environments and positive health among families of CMC. Multicollinearity was assessed using variance inflation factors (VIFs). Variables with VIF values of 9 or greater were considered to exhibit problematic multicollinearity and were excluded from the final models. In the analysis of factors associated with the collaborative environment, service environmental modifications and care improvement modifications were excluded on this basis. Given the relatively limited sample size in relation to the number of predictors included in the models and the constraints of the available dataset, the regression analyses were interpreted as exploratory and intended to examine associations rather than infer causal relationships.

#### 2.7.1. Association Between Collaborative Environment and Environmental Modifications After Implementation

To examine which environmental modifications contribute to a collaborative environment that reduces family burden, a multiple regression analysis using the forced entry method was conducted. The collaborative environment after environmental modifications was set as the dependent variable and the subcategories of environmental modifications were entered as independent variables.

#### 2.7.2. Association Between Positive Health in Families and Environmental Modifications

To identify the environmental modifications associated with higher levels of positive health in families, a multiple regression analysis using the forced entry method was performed. Positive health in families was set as the dependent variable, and the subcategories of environmental modifications were entered as independent variables.

#### 2.7.3. Analysis of Factors Influencing Positive Health in Families

To identify the factors associated with positive health in families, multiple regression analysis using the forced entry method was conducted. The total positive health in families score was set as the dependent variable, and potential influencing factors, including pre-modification family well-being, child characteristics, family characteristics, and service-related factors, were entered as independent variables.

## 3. Results

### 3.1. Descriptive Statistics

A total of 96 responses were collected from families. Of these, 90 responses (93.8%) met the eligibility criteria and were included in the final analysis.

The characteristics of the families and the implementation status of environmental modifications are summarized in Table S4 (Supplementary Materials).

### 3.2. Factors Influencing the Collaborative Environment After Environmental Modifications

A multiple regression analysis was conducted with the total score of the collaborative environment as the dependent variable and the subcategories of environmental modifications as independent variables. The results are presented in Table 1.

Multiple regression analysis demonstrated that family-led environmental modifications (β = 0.670, *p* < 0.001), family-led environmental modifications facilitated by professionals (β = −0.775, *p* = 0.009), and physical environmental modifications (β = 0.679, *p* = 0.015) were significantly associated with the collaborative environment. In contrast, community environmental modifications were not significantly associated with the collaborative environment (β = 0.151, *p* = 0.435).

### 3.3. Associations Between Environmental Modifications and Positive Health in Families

Multiple regression analysis was conducted with the total score of positive health in families as the dependent variable and the subcategories of environmental modifications as independent variables.

Multiple regression analysis demonstrated that family-led environmental modifications were positively associated with positive health in families (β = 0.487, *p* = 0.018). Care improvement modifications were also positively associated with positive health in families (β = 0.597, *p* = 0.031). In contrast, family-led environmental modifications facilitated by professionals (β = −0.486, *p* = 0.088), physical environmental modifications (β = −0.189, *p* = 0.477), and community environmental modifications (β = 0.155, *p* = 0.453) were not significantly associated with positive health in families. Detailed regression results are presented in Supplementary Table S5.

### 3.4. Factors Associated with Positive Health in Families

Multiple regression analysis was conducted with the total score for positive health in families as the dependent variable and potential associated factors as independent variables. The analysis demonstrated that pre-modification family well-being (β = 0.464, *p* < 0.001) and the presence of someone to consult (β = 0.330, *p* = 0.011) were significantly associated with positive health in families. Detailed regression results are presented in Supplementary Table S6.

## 4. Discussion

This study examined the collaborative environment between families and professionals in daily living settings, the environmental modifications related to positive health in families, and their promoting factors. The results showed that family-led and physical environmental modifications were positively associated with the collaborative environment, whereas professional-facilitated family-led environmental modifications were negatively associated. Additionally, family-led environmental modifications were associated with higher levels of positive health in families. Furthermore, pre-modification family well-being and the presence of someone to consult were identified as factors associated with positive health in families. These findings suggest that the collaborative environment is not formed by a single factor but through the interaction of multiple elements, including family involvement, physical living conditions, and the way professionals engage with families.

These findings indicate that family-led environmental modifications are closely related to the formation of a collaborative environment. Daily living settings function as care environments and spaces for family life. Therefore, environmental modifications should be guided by medical considerations and a family-centered care perspective that reflects family values and life contexts [27]. The positive association between family-led modifications and collaborative environments supports the importance of families as key decision-makers in organizing care environments. Families possess contextual knowledge about their daily lives, including routines and roles. Incorporating this perspective may facilitate the integration of medical care and daily life, enabling professional support to become more aligned with real-life situations. Previous studies have also reported that family participation in decision-making and collaborative communication with professionals promote family-centered care [28]. Therefore, family-led environmental modifications may facilitate care coordination grounded in daily life and contribute to the development of collaborative environments in which families and professionals contribute their respective expertise.

Both family-led and physical environmental modifications were associated with the collaborative environment. These modifications involve adjusting physical conditions and continuous interactions between families and professionals as care is adapted to daily life. Thus, establishing a structured home care environment is essential to ensure safe and continuous care [12]. Families are actively involved in organizing their home environment, including equipment placement, supply management, movement flow, and safety measures, thereby creating functional care spaces within daily living environments [29]. Such active involvement may promote information sharing and participation in decision-making and may contribute to the development of collaborative environments.

By focusing specifically on family respondents, this study provides additional insights into how families perceive collaborative environmental modification processes in daily living settings.

By contrast, family-led environmental modifications facilitated by professional roles showed a negative association. However, because this study employed a cross-sectional design, these findings should not be interpreted as indicating that professional involvement negatively affects collaborative environments or family autonomy. Rather, the findings may reflect the complexity and context-dependent nature of collaborative processes in daily living settings. Within shared decision-making frameworks, professionals are expected to provide medical information and structure options, whereas the final decisions should reflect patient and family values [30]. Previous studies have emphasized the central role of family caregivers in decision-making and care coordination within home care settings [31]. Therefore, families may also play leading roles in environmental modifications in daily living settings. However, this does not negate the importance of professional roles. In this study, families of CMC who had well-established collaborative relationships with professionals may have been more likely to actively engage in environmental modifications. In addition, families with greater coping capacity or social support may also have been more likely to actively engage in environmental modifications and report higher levels of positive health. On the other hand, previous studies have reported that families of children with medical complexity experience multidimensional burdens across physical, psychological, and social domains due to the ongoing responsibility of providing complex medical care [7,8]. Therefore, healthcare professionals are required to comprehensively assess the developmental stage of the child, as well as the family’s circumstances and needs, while providing appropriate support that respects family autonomy. In pediatric settings, family-centered care positions families as central partners [32]. These frameworks suggest that professionals play important facilitative roles in supporting family decision-making and collaboration [33]. While this study sought to quantitatively examine family-centered environmental modifications, the nuanced experiences and relational aspects of collaborative care may not have been fully captured through quantitative measures alone. Collaborative care processes in daily living settings are inherently relational, dynamic, and context-dependent. Considering these characteristics, it is important to respect families as primary decision-makers in daily care environments.

However, the negative association observed in this study should be interpreted cautiously. Although the categories were conceptually distinguished according to the primary driving role in environmental modification processes, some conceptual overlap between categories may remain. Initial analyses suggested possible multicollinearity among several predictors; therefore, the regression models were re-examined and revised. Nevertheless, the observed association may partly reflect the conceptual and statistical complexity of categorizing collaborative environmental modification processes, as well as the medically and socially complex situations experienced by families requiring greater professional involvement. These factors may have contributed to the opposite directions of the regression coefficients observed in this study. Therefore, these findings should be interpreted as exploratory associations rather than evidence that professional involvement negatively influences collaborative environments or family autonomy.

This study also found that family-led environmental modifications and care improvement environmental modifications were associated with positive health in families. During the process of environmental modification, families identify challenges and participate in decision-making through dialogues with professionals. This process may strengthen internal resources, such as problem-solving and decision-making abilities [34,35,36]. In addition, when families collaborate with professionals to improve care and experience meaningful changes within daily life, this process may enhance self-efficacy and a stronger sense of agency. However, because this study employed a cross-sectional design, it cannot be concluded that environmental modifications directly improved positive health. Rather, families with higher baseline well-being or coping capacity may have been more likely to actively engage in environmental modifications. Therefore, these associations should be further examined through longitudinal or interventional studies. Thus, family-led environmental modifications can be understood not only as functional adjustments to the care environment but also as a dynamic process that strengthens family capacities and supports positive health.

Furthermore, pre-modification family well-being and the presence of someone to consult were associated with positive health in families. These findings suggest that existing psychological and social resources function as a foundation for positive health. Particularly, having someone to consult may provide emotional and informational support, reduce isolation, and promote adaptive coping. Recent studies have shown that social support buffers caregiver stress and promotes psychological well-being among family caregivers [37,38]. The present findings further support its importance for positive health in families.

The association with pre-modification well-being indicates that positive health may develop over time through accumulated life experiences, rather than through isolated interventions. Therefore, professional support for environmental modification may involve the assessment and strengthening of families’ existing internal and external resources. An additional consideration is that medical complexity should not be understood solely as a function of disease severity or the amount of medical care required. Emerging evidence suggests that medical complexity and nursing complexity are related but distinct constructs [39,40]. Children with similar clinical conditions may require different levels of caregiving coordination and support depending on family resources, environmental circumstances, and available support systems. This perspective may help explain why family-led environmental modifications, collaborative environments, and social resources were associated with positive health in families beyond the child’s medical condition alone. To promote positive health in families, it is important to support environmental modifications as well as assess and enhance families’ well-being and social networks.

Overall, this study demonstrated that collaborative environments in daily living settings are shaped by the interactions between family-led environmental modifications, professional engagement, and physical environmental conditions. Importantly, families are the primary decision-makers in these settings. While professionals play supportive and coordinating roles, their essential function is to facilitate family autonomy, rather than replacing it. These findings highlight the need to reframe nursing practice in community-based care for CMC, shifting from a sole focus on safety and technical support to one that prioritizes family-centered decision-making and the activation of family resources. Strengthening family-centered decision-making may be the key to sustaining both collaborative environments and positive health in families.

This study has several limitations. First, the indirect recruitment approach and the inability to determine the exact number of eligible families who received the questionnaires or survey links may have introduced selection bias. Second, the relatively small sample size and use of complete-case analysis may have reduced statistical power; therefore, the findings should be interpreted cautiously as exploratory in nature. Furthermore, although variables with substantial multicollinearity were excluded from the final models, several retained predictors demonstrated moderate levels of multicollinearity, which may have affected the stability and precision of the regression coefficients. In addition, residual confounding cannot be ruled out because we were unable to fully adjust for important factors such as the child’s clinical severity, socioeconomic status, caregiver education, duration of home care, and technology dependence. Third, because this study employed a cross-sectional design and self-reported questionnaire data, causal relationships cannot be inferred, and the findings may have been influenced by social desirability bias and common-method variance. Families with greater baseline resources, coping capacity, or social support may have been more likely to undertake environmental modifications and report higher levels of positive health. Therefore, reverse causality should also be considered when interpreting the findings. Fourth, data were based on participants’ recall of environmental modifications, which may be subject to recall bias. Fifth, the study was conducted in a limited number of facilities in Japan, which may restrict generalizability to other contexts.

Additionally, because the measures used in this study were newly developed based on concept analysis and qualitative interview research, further psychometric evaluation, including construct validity, factor structure testing, and validation in families of children with medical complexity, is needed in future studies.

Future studies should replicate these findings with larger and more diverse samples and consider longitudinal or interventional designs to examine the long-term relationships between family- centered environmental modifications and positive health. Furthermore, because this study employed a quantitative cross-sectional design, the nuanced experiences and contextual perspectives of families may not have been fully captured. Future qualitative or mixed-methods studies are needed to better understand how families experience collaborative environmental modifications in daily living settings.

Despite these limitations, the findings provide important implications for nursing practice in supporting the ongoing care of CMC in community settings. Nurses should integrate safety management with family-centered dialogue to understand families’ desired lifestyles and collaboratively plan value-based environmental modifications. These modifications should be viewed not merely as physical adjustments, but as processes that support family participation in decision-making. The findings suggest that healthcare professionals play facilitative roles in supporting family autonomy. In addition, assessing and strengthening family social networks may help sustain the effects of environmental modifications.

## 5. Conclusions

The findings of this study have several important implications for nursing practice in supporting the continuation of community-based care for CMC.

First, nurses should go beyond arranging medical equipment and ensuring safety, particularly during pre-discharge visits and the early transition to home care. It is important to engage in dialogue with families to understand their preferred ways of living and collaboratively plan environmental modifications that reflect their values. Environmental modifications should not be viewed solely as physical adjustments but also as processes that support family participation in decision-making.

Second, although professionals play key roles in providing knowledge and coordinating resources, their primary function is not to replace family autonomy but to facilitate and support it.

Third, considering the association between positive health in families and the presence of someone to consult, it is important to assess families’ social networks as a key component of care. Identifying accessible sources of emotional and informational support and connecting families to appropriate resources when needed may help maintain the benefits of environmental modifications.

This study employed a cross-sectional design and therefore could not establish causal relationships. Future longitudinal studies are required to examine the causal relationships between environmental modification processes and positive health in families.

## Figures and Tables

**Table 1:** Associations between environmental modifications and the collaborative environment.

Independent Variable: Environmental Modifications	B	SE	95% CI	Standardized Coefficient (β)	*p*-Value	Tolerance	VIF	Adjusted R^2^
Family-led environmental modifications (total)	2.084	0.572	0.925–3.244	0.670	<0.001 **	0.436	2.295	0.471
Family-led environmental modifications facilitated by professionals (total)	−1.073	0.389	−1.861–−0.285	−0.775	0.009 **	0.186	5.363	
Physical environmental modifications (total)	2.085	0.818	0.426–3.744	0.679	0.015 *	0.207	4.829	
Community environmental modifications (total)	0.563	0.713	−0.883–2.009	0.151	0.435	0.400	2.499	

Note. Dependent variable: total score for the collaborative environment after environmental modifications. Results are based on multiple regression analysis. * *p* < 0.05; ** *p* < 0.01. Participants with missing values were excluded from the regression analyses; therefore, the analytic sample size was 41. Service environmental modifications and care improvement modifications were excluded from the model because of multicollinearity (VIF ≥ 9).

## Data Availability

The dataset is available on reasonable request from the corresponding author due to ethical restrictions.

## Supplementary Materials

The following supporting information can be downloaded at: https://www.mdpi.com/article/10.3390/nursrep16060192/s1, Figure S1. Participant flow diagram. Table S1. Items Related to the Care Environment. Table S2. Items Related to Environmental Modifications. Table S3. Measurement Instruments and Psychometric Properties. Table S4. Participant Characteristics. Table S5. Associations between environmental modifications and positive health in families. Table S6. Factors associated with positive health in families.

## Public Involvement Statement

No public involvement in any aspect of this research.

## Guidelines and Standards Statement

This manuscript was drafted against the STROBE checklist for cross-sectional research.

## Abbreviation

The following abbreviation is used in this manuscript:

CMC	Children with medical complexity
