# Supplementary material for: Associations of Environmental Modifications and Collaborative Care Environments with Positive Health in Families of Children with Medical Complexity: A Secondary Analysis
Source: Nurs Rep. 2026 Jun 5;16(6):192. doi: 10.3390/nursrep16060192 (PMC13304894; doi:10.3390/nursrep16060192)
Supplement: Supplementary file 1 [file nursrep-16-00192-s001.zip › Figure S1. Participant flow diagram.pdf]

Figure S1. Participant flow diagram

Facilities and associations contacted

- Home-visit nursing stations (n = 500)
- Consultation support offices (n = 150)
- Child development support and after-school day service facilities for CMC (n = 150)
- Family associations (n = 26)

↓

Estimated paper-based questionnaires distributed through facilities

- Home-visit nursing stations: 500 × 1
- Consultation support offices: 150 × 1
- Child development support and after-school day service facilities for CMC: 150 × 2

Estimated total distributed through facilities:

n = 950

Note:

The number distributed through family

associations could not be determined.

↓

Returned questionnaires/responses

n = 96

↓

Excluded questionnaires

n = 6

Reasons:

- No environmental modifications implemented
- Incomplete responses
- Missing data

↓

Final analytic sample

n = 90
