# Supplementary material for: Associations of Environmental Modifications and Collaborative Care Environments with Positive Health in Families of Children with Medical Complexity: A Secondary Analysis
Source: Nurs Rep. 2026 Jun 5;16(6):192. doi: 10.3390/nursrep16060192 (PMC13304894; doi:10.3390/nursrep16060192)
Supplement: Supplementary file 1 [file nursrep-16-00192-s001.zip › Table S2. Items Related to Environmental Modifications.pdf]

Table S2. Items Related to Environmental Modifications

**Physical modifications**：Adjustments of physical space, supplies, and preparedness to support daily care, safety, and child development in living settings.	**Agent**
Families procured supplies necessary for the child's care and upbringing	F
Families structured the environment to promote the child's growth and development	F
Families and professionals confirmed with each other the preparations needed to provide care for the child in daily living settings	F&P
Families and professionals collaborated to adjust the environment to promote the child's physical and mental growth and development	F&P
Professionals supported families in maintaining their living space	P
Professionals supported families in preparing for emergencies and disasters	P
**Family-led environmental modifications**：Modifications initiated and directed by families based on their values, preferences, daily routines, and childrearing priorities.	
Families made decisions regarding the child's medical care and upbringing	F
Families cooperated so that all family members could be involved with the child	F
Families acquired medical care and childrearing skills at their own pace	F
Families adjusted the timing of the child's care while considering the overall life of the family	F
Families made efforts to manage their own physical and mental health	F
Families sought to enhance their motivation for childrearing	F
Families were aware of their social roles	F
**Family-led environmental modifications facilitated by professionals:** Family-directed modifications supported by professionals through informational, consultative, coordinative, or facilitative support.	
Families consulted professionals about concerns and difficulties related to the child's health and upbringing	F
Families asked professionals about things they did not understand based on the information provided	F
Families communicated their thoughts about the child and their views on childrearing to professionals	F
Families gathered information on local services and social resources	F
Families obtained information about the necessary financial support	F
Families prepared for responses to emergencies and disasters	F
Families reported to professionals about changes resulting from service use	F
Families and professionals discussed the kind of life and childrearing the family desired	F&P
Families and professionals discussed childrearing approaches that made use of the family's life circumstances and background	F&P
Families and professionals discussed the healthy growth and development of siblings	F&P
Families and professionals collaborated in providing care for the child	F&P

Families and professionals discussed challenges in childrearing	F&P
Professionals supported the decision-making of the child and family	P
**Community environmental modifications:** Modifications aimed at promoting children's and families' participation, connection, and inclusion in the local community.	
Families advocated for making the community more livable for the child and family	F
Families accepted the child's connection with the community	F
Professionals supported the child and family in building connections with the community	P
Professionals engaged with the community to make it more accommodating for the child and family	P
**Service environmental modifications:** Modifications related to accessing, coordinating, and optimizing health, welfare, and community services for family life and childrearing.	
Families utilized services	F
Families and professionals collaborated to develop comprehensive care plans for community living	F&P
Professionals supported families in achieving their desired lifestyle and childrearing goals	P
Professionals respected family life in the course of providing support	P
Professionals supported families in selecting necessary services	P
Professionals provided information to reduce families' financial burden	P
Professionals collaborated with other relevant disciplines involved in the child and family's care	P
Professionals supported families in assessing the child's condition	P
Professionals supported families in maintaining and promoting their health	P
Professionals developed systems for providing necessary community services	P
Professionals continued learning to improve the quality of support for children requiring medical care	P
**Care improvement environmental modifications:** Modifications aimed at improving the quality, responsiveness, and family-centeredness of care and support provided to children and families.	
Families and professionals discussed challenges related to services	F&P
Professionals provided support with consideration for the family's acceptance process, physical and mental condition, and relationships	P
Professionals provided information using terminology that was easy for families to understand	P
Professionals actively addressed families' concerns and difficulties	P
Professionals supported families in working together to provide childrearing and care	P
Professionals provided guidance on medical care and childrearing tailored to the family	P
Professionals supported families in adjusting the child's living environment	P

Note. Agents of environmental modifications were conceptually categorized according to the primary driving role in the modification process: Family-led (F); Jointly led by families and professionals (F&P); Professionally facilitated (P).
