# Supplementary material for: Associations of Environmental Modifications and Collaborative Care Environments with Positive Health in Families of Children with Medical Complexity: A Secondary Analysis
Source: Nurs Rep. 2026 Jun 5;16(6):192. doi: 10.3390/nursrep16060192 (PMC13304894; doi:10.3390/nursrep16060192)
Supplement: Supplementary file 1 [file nursrep-16-00192-s001.zip › Table S3. Measurement Instruments and Psychometric Properties.pdf]

Table S3. Measurement Instruments and Psychometric Properties

Scale	Number of items	Score range	Cronbach's α
Care Environment	35	0-210	.96
Environmental Modifications	48	48-240	.96
● Physical environmental modifications	6	6-30	.74
● Family-led environmental modifications	7	7-35	.75
● Family-led environmental modifications facilitated by professionals	13	13-65	.91
● Community environmental modifications	4	4-20	.67
● Service environmental modifications	11	11-55	.94
● Care improvement modifications	7	7-35	.93
Family well-being	13	0–78	.92
Positive health	10	10–50	.88

Note: The environmental scale consisted of 35 items rated on a 7-point Likert scale (0–6), with a theoretical total score range of 0–210. Items marked as "not applicable" were treated as missing values and excluded from the calculation of total scores, resulting in varying denominators across participants. The environmental modification scale consisted of 48 items rated on a 5-point Likert scale (1–5), with a theoretical total score range of 48–240. Responses of "not necessary" and "don't know" were treated as missing values and excluded from the calculation of total scores, resulting in varying denominators across participants.
