# Supplementary material for: Associations of Environmental Modifications and Collaborative Care Environments with Positive Health in Families of Children with Medical Complexity: A Secondary Analysis
Source: Nurs Rep. 2026 Jun 5;16(6):192. doi: 10.3390/nursrep16060192 (PMC13304894; doi:10.3390/nursrep16060192)
Supplement: Supplementary file 1 [file nursrep-16-00192-s001.zip › Table S4. Participant Characteristics.pdf]

Table S4. Participant Characteristics

Family characteristics N=90

Item		Value
Primary caregiver (multiple responses))	Mother	87(96.7%)
	Father	12(13.3%)
	Grandparent(s)	4(4.4%)
	Other	1(1.1%)
Age (CMC) (n = 71)	mean ± SD	3.5 ± 4.1
Presence of siblings	Yes	65(72.2%)
	No	25(27.8%)
Number of siblings (n = 65)	1	43(47.8%)
	2	12(13.3%)
	3 or more	10(15.4%)
Employment status of primary caregiver	Yes	44(48.9%)
	No	46(51.1%)
Interaction with extended family	Always/Sometimes	58(64.4%)
	Rarely/Never	32(35.6%)
Presence of a person to consult with	Yes	76(84.4%)
	No	14(15.6%)
Family relationships (n = 89)	Good/Somewhat good	85(95.5%)
	Somewhat poor/Poor	4(4.5%)

Environmental modification case characteristics

Item		N（%）
Primary diseases of CMC	Neurological, gastrointestinal, and respiratory disorders	63(70.0%)
	Neurological and gastrointestinal disorders	5(5.6%)
	Neurological, gastrointestinal, and excretory disorders	3(3.3%)
	Neurological and respiratory disorders	1(1.1%)
	Neurological, gastrointestinal, excretory, and respiratory disorders	1(1.1%)
	Gastrointestinal and respiratory	4(4.4%)

	disorders	
	Gastrointestinal disorders	5(5.6%)
	Gastrointestinal and excretory	3 (3.3%)
	Respiratory disorders	3(3.3%)
	Excretory disorders	2(2.2%)
Context of environmental modification	Preparation for transition to daily living	46(51.1%)
	Change in CMC care methods	13(14.4%)
	Change in CMC health condition	13(14.4%)
	CMC life events	9(10.0%)
	Family health problems	4(4.4%)
	Family life events	1(1.1%)
	Sibling life events	1(1.1%)
	Other	3(3.3%)
Child's health status	Stable	28(31.1%)
	Unstable	62(68.9%)
Child's signs and responses	Readable	16(17.8%)
	Difficult to read	74(82.2%)
Child's expression of intent	Understandable	19(21.1%)
	Not understandable	71(78.9%)
Desired services	Present	47(52.2%)
	Absent	30(33.3%)
	Not applicable to services	13(14.4%)
Service accessibility	Easy to use	29(32.2%)
	Difficult to use	48(53.3%)
	Not applicable to services	13(14.4%)
Information about necessary services	Sufficient/Some	38（42.2%）
	Little/None	52（57.8%）
People the family communicates with	Present	75(83.3%)
	Absent	13(14.4%)
	Unknown	2（2.2%）
People the family can ask for help	Present	54(60.0%)
	Absent	34(37.8%)
	Unknown	2（2.2%）
People the family can share private matters with	Present	57(63.3%)
	Absent	31(34.4%)

Unknown	2（2.2%）

Note: Missing values were excluded from the denominator; therefore, denominators vary across variables. Categorical variables are expressed as number (%), and continuous variable (age) is expressed as mean ± standard deviation.
