# Supplementary material for: Associations of Environmental Modifications and Collaborative Care Environments with Positive Health in Families of Children with Medical Complexity: A Secondary Analysis
Source: Nurs Rep. 2026 Jun 5;16(6):192. doi: 10.3390/nursrep16060192 (PMC13304894; doi:10.3390/nursrep16060192)
Supplement: Supplementary file 1 [file nursrep-16-00192-s001.zip › Table S5. Associations between environmental modifications and positive health in families.pdf]

Table S5. Associations between environmental modifications and positive health in families

Independent variables: Environmental modification subcategories	B	SE	95% CI	Standardized coefficient (β)	*p*-value	tolerance	VIF	Adjusted R^2
Family-led environmental modifications (total)	.856	.348	0.154–1.559	.487	.018*	.431	2.319	
Family-led environmental modifications facilitated by professionals (total)	−.366	.210	−0.790–0.057	-.486	.088	.218	4.597	
Physical environmental modifications (total)	−.355	.494	−1.352–0.642	-.189	.477	.243	4.123	.273
Community environmental modifications (total)	.318	.420	−0.530–1.165	.155	.453	.400	2.499	
Care improvement modifications (total)	.712	.320	0.067–1.357	.597	.031*	.235	4.262	

Note: Dependent variable: Total score of positive health in families. Results are based on multiple regression analysis; * $p < .05$. Participants with missing values were excluded from the regression analyses; therefore, the analytic sample size was 49. Service environmental modifications was excluded from the model because of multicollinearity (VIF ≥ 9).
