# Supplementary material for: Associations of Environmental Modifications and Collaborative Care Environments with Positive Health in Families of Children with Medical Complexity: A Secondary Analysis
Source: Nurs Rep. 2026 Jun 5;16(6):192. doi: 10.3390/nursrep16060192 (PMC13304894; doi:10.3390/nursrep16060192)
Supplement: Supplementary file 1 [file nursrep-16-00192-s001.zip › Table S6. Factors associated with positive health in families.pdf]

Table S6. Factors associated with positive health in families

Independent variables: Factors influencing positive family health	B	SE	95% CI	Standardized coefficient (β)	*p-value*	tolerance	VIF	Adjusted R^2
Pre-modification family well-being	.246	.060	0.127–0.366	.464	< .001***	.871	1.148	
Occurrence of sudden changes in the child's condition	−1.849	1.878	−5.618–1.920	−.108	.329	.915	1.093	
Interaction with other families	.199	1.007	−1.821–2.219	.024	.844	.719	1.391	
Presence of someone to consult	7.079	2.679	1.704–12.454	.330	.011*	.709	1.410	
Employment status of the primary caregiver	−.323	1.927	−4.190–3.544	−.020	.868	.792	1.263	.316
Availability of desired services	−1.747	2.127	−6.016–2.521	−.103	.415	.699	1.430	
Ease of access to services	3.475	2.114	−0.766–7.717	.205	.106	.708	1.412	
Presence of relatives or friends to stay in contact with	−1.297	2.765	−6.845–4.251	−.058	.641	.718	1.392	
Presence of relatives or friends to seek help from	1.730	2.384	−3.054–6.514	.105	.471	.527	1.898	
Presence of relatives or friends to talk to about private matters	2.231	2.340	−2.466–6.927	.135	.345	.552	1.811	

Note: Dependent variable: Total score of positive health in families. Results are based on multiple regression analysis; * $p < .05$, *** $p < .001$. Participants with missing values were excluded from the regression analyses; therefore, the analytic sample size was 63.
